# Primary malignant mixed Müllerian tumor arising from the mesorectum with a synchronous ovarian cancer: a case report and review of the literature

**DOI:** 10.1186/1752-1947-5-15

**Published:** 2011-01-18

**Authors:** Chuang-Chi Huang, Cheng-Jen Ma, Wan-Ting Huang, Te-Fu Chan, Jaw-Yuan Wang

**Affiliations:** 1 Department of Surgery, Kaohsiung Medical University Hospital, Kaohsiung Medical University, Kaohsiung, Taiwan; 2 Department of Pathology, Kaohsiung Medical University Hospital, Kaohsiung Medical University, Kaohsiung, Taiwan; 3 Departments of Obstetrics and Gynecology, College of Medicine, Kaohsiung Medical University, Kaohsiung, Taiwan; 4 Graduate Institute of Medicine, College of Medicine, Kaohsiung Medical University, Kaohsiung, Taiwan; 5 Departments of Obstetrics and Gynecology, Kaohsiung Medical University Hospital, Kaohsiung Medical University, Kaohsiung, Taiwan; 6 Department of Surgery, Faculty of Medicine, College of Medicine, Kaohsiung Medical University, Kaohsiung, Taiwan; 7 Graduate Institute of Medical Genetics, College of Medicine, Kaohsiung Medical University, Kaohsiung, Taiwan; 8 Cancer Center, Kaohsiung Medical University Hospital, Kaohsiung, Taiwan

## Abstract

**Introduction:**

Extragenital malignant mixed Müllerian tumor is an extremely rare presentation of malignant mixed Müllerian tumor, especially when combined with a synchronous ovarian cancer.

**Case presentation:**

We report the clinical course and pathologic findings of a case of mesorectal malignant mixed Müllerian tumor with synchronous ovarian cancer, in a 50-year-old, gravida 0, para 0, Han Chinese woman with regular menstruation. This is the sixteenth case in the English literature of extragenital malignant mixed Müllerian tumor combined with synchronous or metachronous malignancy reported.

**Conclusion:**

Although extragenital malignant mixed Müllerian tumor is very rare and has a poor prognososis, a longer survival time might be achieved with treatment by cytoreductive surgery, radiotherapy and chemotherapy.

## Introduction

Malignant mixed Müllerian tumor (MMMT) is an uncommon tumor in females and the occurrence of this disease outside the genital tract is extremely rare. In a review of the English literature since 1955, only 48 cases of extragenital MMMT have been reported other than the presented case. Sixteen out of these 49 (32.7%) extragenital MMMTs [1], including this case, were associated with synchronous or metachronous colonic cancer or gynecologic malignancy and serous carcinoma of the peritoneum (Table 1). The MMMT often presents in elderly menopausal women and is a highly aggressive tumor. We report the clinical course and pathologic findings of an extragenital MMMT arising from the mesorectum in a perimenopausal woman and a review of the English literature.

**Table 1:** Previous reports of malignant mixed Müllerian tumor (MMMT) with synchronous or metachronous neoplasm

Case	Year	Author	Age	Primary site	Tissue type	Associated tumor	Treatment	Prognosis
1 [15]	1983	Hermann and Tessler	72	Abdominal retroperitoneum	Heterologous	Ovarian serous papillary carcinoma, metachronous	Operation, CT (Adriamycin (doxorubicin), cytoxan, DTIC, vincristine)	Death at six months

2 [16]	1988	Chen and Wolk	58	Pelvic peritoneum	Homologous	Ovarian serous papillary carcinoma, metachronous	Operation, RT	Death at 11 months

3 [17]	1989	El-Jabbour *et al.*	76	Ascending colon peritoneum	Heterologous	Colonic adenocarcinoma, synchronous	Operation	Death at 14 days

4 [18]	1991	Garde and Jones *et al.*	65	Diaphragmatic peritoneum	Heterologous	Ovarian endometrioid adenocarcinoma, metachronous	Operation, CT (Adriamycin (doxorubicin), cisplatin, ifosfamide)	Death at six months

5 [19]	1991	Solis *et al.*	54	Pelvic peritoneum	Heterologous	Serous carcinoma of peritoneum, synchronous	Operation, CT (Adriamycin (doxorubicin), cisplatin, cytoxan)	Unknown

6 [9]	1994	Garamvoelgyi *et al.*	59	Pelvic peritoneum	Heterologous	Endometrial adenocarcinoma, metachronous	Operation, CT ( ifosfamide)	Death at 24 months

7 [9]	1994	Garamvoelgyi *et al.*	64	Pelvic peritoneum	Homologous	Fallopian tube cacinoma in situ, synchronous	Operation	Death at eight months

8 [9]	1994	Garamvoelgyi *et al.*	84	Retrouterine peritoneum	Heterologous	Colonic adenocarcinoma, synchronous	Operation	Death at two months from heart disease

9 [20]	1995	Mira *et a*l.	62	Pelvic peritoneum	Heterologous	Ovarian endometrioid adenocarcinoma, metachronous	Operation	Survival for 28 months

10 [21]	1997	Rose *et al.*	71	Peritoneum	Homologous	Uterine cervical adenocarcinoma, synchronous	Operation, CT (cisplatin, ifosfamide)	Death at six months

11 [22]	2001	Shen *et al.*	33	Pelvic peritoneum	Heterologous	Endometrial adenocarcinoma, metachronous	Operation	Death at 12 months

12 [22]	2001	Shen *et al.*	40	Pelvic	Heterologous	Fallopian tube carcinoma, metachronous	Operation	Unknown

13 [23]	2005	Mikami *et al.*	53	Mesentery	Heterologous	Fallopian tube carcinoma, metachronous	Operation, CT	Survival for six months

14 [24]	2005	Shaco-Levy	85	Omentum	Heterologous	Colonic adenocarcinoma, metachronous	Operation	Survival for three months

15 [1]	2006	Ma *et al.*	62	Mesentery	Homologous	Ovarian adenocarcinofibroma, synchronous	Operation, CT (ifosfamide, carboplatin, etoposide)	Death at 30 months

16	2008	Current case	50	Mesentery	Homologous	Ovarian adenocarcinoma, synchronous	Operation	Death at 10 months

CT, computed tomography; DTIC, Dacarbazine; RT, radiotherapy

## Case presentation

**Figure 1:**
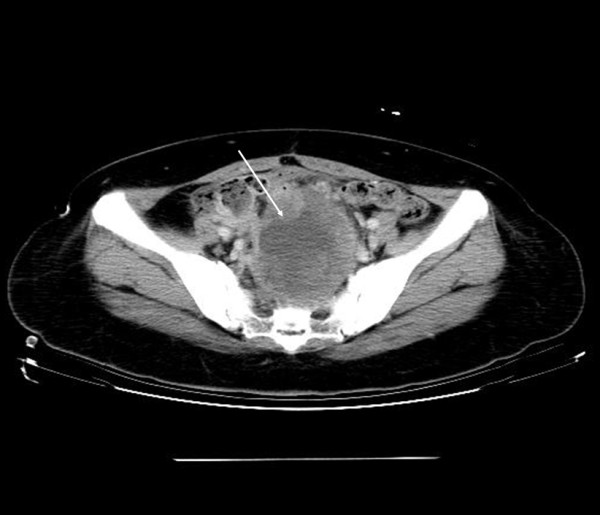
**A large low-density mass lesion was noted in the pelvic cavity and a significant mass effect at rectosigmoid and bladder was also noted (arrow)**.

The patient case was a 50-year-old, gravid 0, para 0 (G0P0), unmarried Han Chinese woman with regular menstruation. Six months ago, she visited another medical center in Southern Taiwan for abdominal bloating, where bilateral ovarian tumors were diagnosed. At laparotomy, a left ovarian cystic tumor (35 × 20 × 10 cm) and a right ovarian tumor (12 × 8.5 × 6 cm) with normal uterus and cervix were noted. An additional tumor of about 12 × 9 × 8 cm in size was also found in the mesorectum of the rectosigmoid colon. Resection of the mesorectum and bilateral oophorectomy was performed at the first operation at another medical center. The histopathology report revealed bilateral ovarian cancer (endometrioid adenocarcinoma) and malignant mixed Müllerian tumor from the mesorectum with biphasic differentiation (adenocarcinomatous and spindle cell sarcomatous elements). No heterologous element was identified. No further treatment was performed after the first time of operation. However, she felt progressive abdominal bloating and dysuria recently. She, therefore, visited the department of surgery of our hospital. On physical examination a lower abdominal mass was palpated. An abdominal computed tomography scan revealed a large low density mass in the pelvic cavity (Figure 1). The maximum size of this lesion was about 15 cm in its long-axis diameter. This mass affected the bladder and the rectosigmoid colon. Laboratory tests showed that the serum lactate dehydrogenase level was 271 IU/L. The serum CA 125 level was elevated up to 154.3 U/mL, while the serum CA19-9 level was within the normal range.

**Figure 2:**
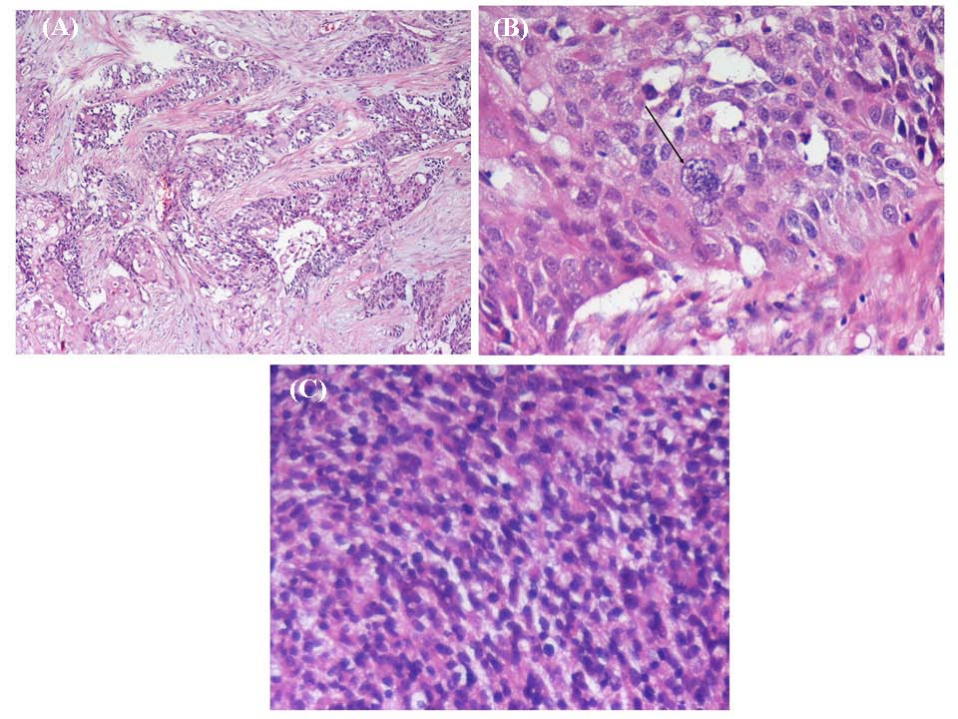
**(A) These cells with local glandular and squamoid differentiation were noted in the carcinomatous component (100×)**. (B) The bizarre tumor giant cell was noted in the carcinomatous component (arrow; 400x). (C) The patternless oval to spindled neoplastic cells was noted in the sarcomatous component (200x).

On suspicion of the recurrence of a tumor, another laparotomy was performed. The pelvic cavity was fully occupied by a huge cystic mass with adjacent organ involvement. A tumor measuring 12 × 10 × 8 cm arising from the mesorectum was identified - the terminal ileum was also involved. The tumor infiltrated into the pelvic floor and the retroperitoneum and a palliative resection of the rectosigmoid colon with an end-to-end anastomosis was performed. Unfortunately, 10 days later, the patient had an anastomotic leakage caused by the penetration of the drain tube which was noted when a colonoscopy was performed. Consequently, an ileostomy was constructed for fecal diversion as the healing of the leakage site had failed. The pathologic findings showed neoplastic cells with areas of local glandular and squamoid differentiation. In addition, bizarre giant tumor cells in the carcinoma component were also noted (Figure 2A and 2B). Patternless oval to spindled neoplastic cells were noted in the sarcoma component (Figure 2C). Immunohistochemical studies showed that CK7 and CD10 staining were positive but that the CK20 staining was negative. After one and a half months in our department, she recovered uneventfully and was transferred to the division of medical oncology for chemotherapy. Chemotherapy, with regimen of bleomycin, etoposide and cisplatin, was arranged but pancytopenia with nosocomial infection was noted after the chemotherapy. Due to the poor response to systemic chemotherapy, hospice care was suggested and she was referred to the previous medical center.

## Discussion

MMMT arising from the female genital tract is a rare disease, comprising less than 1% of all gynecological malignancies, and MMMT of extragenital origin is even rarer. MMMT arises from the Müllerian system which develops to form the fallopian tubes, uterus and the upper portion of the vagina and often occurs in menopausal women. Since histological evaluation shows both carcinoma (epithelial) and sarcoma (mesenchymal) components, this disorder is also named carcinosarcoma. MMMT is classified into homologous or heterologous according to the sarcomatous component. Extragenital MMMT can occur at any site of peritoneum and is one type of primary peritoneal carcinomas (PPC) which was first described by Swerdlow in 1959 [2]. It has the characteristics of involvement in the peritoneum by carcinoma without an obvious primary site [3].

The majority of PPCs present in pathology as serous papillary carcinomas, as well as peritoneal mixed epithelial carcinomas, while the extragenital MMMTs are rarely reported. PPC is a rare cancer closely related to epithelial ovarian cancer and develops in cells from the lining of the pelvis and abdomen (peritoneum). These cells are similar to the cells on the surface of the ovaries. Like ovarian cancer, PPC tends to spread along the surface of the pelvis and abdomen. Symptoms of patients with PPC are similar to those with ovarian cancer, including abdominal pain or bloating, nausea, vomiting, indigestion and change in bowel habits. Women with PPC are usually treated similarly to those with widespread ovarian cancer. The therapeutic modalities include cytoreductive surgery as much as possible, followed by the same chemotherapy regimen administrated for ovarian cancer. Look *et al.* asserted that optimal cytoreduction could significantly improve the prognosis of patients [4]. However, PPC is of multifocal origin, which is in contrast to ovarian cancer, and usually infiltrates the peritoneal lining surface. Consequently, cytoreductive surgery is not always optimal and this therapeutic modality needs to be evaluated in order to determine whether it is an appropriate treatment for PPC.

Most PPCs are serous papillary adenocarcinomas with a relatively good prognosis but the primary peritoneal MMMT, a rare type of PPC, usually has an unfavorable outcome according to the previous literature [5]. MMMT of extragenital origin was first reported by Ober and Black in 1955 [6] and, until now, only 48 cases have been reported in the English literature. It has been reported to have arisen from the peritoneum, mesentery, omentum, spleen, diaphragm and retroperitoneum. Among all the reported cases, the majority were menopausal women with a median age of 62.8 years (range 33-87 years). Sixteen of the 49 patients (32.7%) presented with synchronous or metachronous malignancies including colonic (three cases), ovarian (six cases including the present case), fallopian tubal (three cases), endometrial (two), cervical (one) and one synchronous serous carcinoma of the peritoneum. Due to a high incidence of synchronous or metachronous colonic cancer or gynecologic malignancy originating from the Müllerian duct, clinicians should carefully check the genital tract in detail during the resection of primary MMMT.

Little information about the management of extragenital MMMT is available. All suggestions for the treatment extragenital MMMT are based on individual cases. Treatments including cytoreductive surgery and chemotherapy have been reported. Surgical management is usually mandatory due to the clinical presentation caused by the mass effect. However, a radical surgical treatment is often obtained with difficulty. It seems that chemotherapy is more important than surgical treatment and the treatment choice of MMMT is similar to that of genital MMMT.

There are several reports regarding platinum-based chemotherapy activity against MMMT of the ovary. Simon *et al.* reported a patient with MMMT of the ovary who had a suboptimal response to single-agent cisplatin chemotherapy but who demonstrated a complete response with ifosfamide, mesna, Adriamycin (doxorubicin) and dacarbazine [7]. Paclitaxel/carboplatin (PC) or platinum/ifosfamide (PI) has been used for the chemotherapy of ovarian MMMT [8]. The median survival time of patients receiving PC was 19 months. One patient receiving PC as first-line treatment demonstrated a complete response and was free of disease after 33 months. The median survival time of patients managed with PI was 23 months. Three patients with suboptimal disease demonstrated complete response after receiving PI. This study showed the potential activity of PC in MMMT of the ovaries should be further explored.

The role of radiotherapy remains controversial. When a patient presents with a grossly residual tumor, radiotherapy may be considered. Garamvoelgyi *et al.* reported a patient who received postoperative radiotherapy and survived for eight months [9]. Conversely, other authors consider that extragenital MMMT is one kind of PPC and is similar to ovarian epithelial tumor. Muller *et al.* reported six cases of metastasized MMMTs receiving cytoreductive surgery plus intraperitoneal hyperthermic perfusion and adjuvant treatment of CDDP (cis-diamminedichloroplatinum), mitomycin and ifosfamide applied via intraaortic catheter [10]. Four patients were found with no evidence of disease after two, four, 14, and 19 months, respectively. Thus, complete cytoreduction plus hyperthermic peritoneal perfusion plus adjuvant chemotherapy seems to be an effective treatment for recurrent or metastatic MMMT.

A similar case of MMMT of mesenteric origin was reported by Ma *et al.*[1]. The patient died of extensive metastasis 30 months after the diagnosis of MMMT. She received six courses of chemotherapy, including ifosfamide, VP-16 and carboplatin, as well as eight courses of Phyxol (paclitaxel) and cisplatin.

Recently, it has been demonstrated that the presence of BRCA mutations may predispose to primary peritoneal cancers and this neoplasm could be a part of the hereditary breast and ovary cancer syndrome [11]. Immunohistochemical studies, it is suggested that expression of CD10 should be examined - it may be one of the characteristics of MMMT [12,13]. However, the significance of CD10 expression needs to be elucidated by further studies. Our patient's tumor also had an expression of CD10. Regarding the histological component in MMMT, Ozguroglu *et al.* investigated the role of carcinomatous and sarcomatous components on the response to chemotherapy and disease outcome. It also observed that patients with a predominating carcinomatous component had a higher therapeutic response rate (87.5%) than those with a predominating sarcomatous component (66.6%) [14].

## Conclusion

Extragenital MMMT is extremely rare and has a poor prognosis due to its aggressive biological behavior. Synchronous or metachronous gynecologic tumors often exist and a detailed examination of the genital tract must be made before and during the operation. Moreover, improved survival times would probably be obtained if accurate diagnoses and aggressive treatment, including cytoreductive surgery and chemotherapy, are applied early

## Abbreviations

MMMT: malignant mixed Müllerian tumor; PC: paclitaxe/carboplatin; PI: platinum/ifosfamide; PPC: primary peritoneal carcinomas.

## Consent

Written informed consent was obtained from the patient for publication of this case report and any accompanying images. A copy of the written consent is available for review by the Editor-in-Chief of this journal.

## Competing interests

The authors declare that they have no competing interests.

## Authors' contributions

CCH drafted the article. CJM analyzed and interpreted the patient data. WTH photographed and interpreted the pathologic findings. TFC took part in the critical revision. and JYW took part in the surgical approach and final approval of the manuscript. All authors have made substantive intellectual contributions to this study and to the manuscript and have read and approved the final manuscript.
